# Leveraging the Potential of Sorghum as a Healthy Food and Resilient Crop in the South African Food System

**DOI:** 10.3389/fsufs.2022.786151

**Published:** 2022-05-20

**Authors:** Laura M. Pereira, Corinna Hawkes

**Affiliations:** 1 Stockholm Resilience Centre, Stockholm University, Stockholm, Sweden; 2 Centre for Food Policy, City University of London, London, United Kingdom; 3 Global Change Institute, University of the Witwatersrand, Johannesburg, South Africa

**Keywords:** food systems, sorghum, South Africa, indigenous food, healthy and sustainable diets

## Abstract

An erosion of indigenous and traditional foods in the Global South has dramatically changed the global food system in the last 50 years. Reinvigorating these crops and the agro-biodiversity that they represent could provide benefits for healthier and more sustainable food systems. In South Africa, it has been proposed that studying indigenous plants more extensively and focussing on innovation to include them as mainstream foods on people’s plates could improve food and nutrition security. With this background, this paper aims to contribute to addressing this challenge by researching sorghum (*Sorghum bicolor*) to identify the opportunities for innovating around sorghum as a healthy food and resilient crop. The paper traces sorghum through various encounters across the South African food system. The results point at clear areas where policy interventions could bolster the sorghum value chain. These include zero-rating VAT on sorghum products, investing more extensively in research and marketing across diverse stakeholders, raising awareness about the health benefits of sorghum and using public procurement as a way of instigating a market for novel sorghum products. The outcomes of a successful sorghum innovation programme could improve smallholder farmers’ livelihoods, make a healthy food more accessible to South Africans and develop a local market for innovative products that utilize a crop that is resilient to projected climatic changes.

## Introduction

There have been widespread calls to align food systems more closely with nutrition and sustainability goals (Willett et al., 2019). Poor quality diets are a significant cause of ill health worldwide (Afshin et al., 2019). The diets of people living in poverty are typically monotonous and inadequately diverse, dominated by refined cereals; everywhere in the world, industrially produced foods low in nutrients and high in fats and sugars are associated with increasing levels of obesity and diet-related non-communicable diseases like diabetes (Hawkes, 2006; Popkin et al., 2012). The current food system also has an unsustainable level of environmental impact such as biodiversity loss from land use change processes like deforestation, overfishing, high levels of water extraction and greenhouse gas emissions (Gordon et al., 2017).

While food systems are currently not adequately supporting healthy diets and environmental sustainability, they also contain numerous opportunities for leverage points and agents of change (HLPE, 2017). As articulated by the HLPE, “food systems are sprawling networks of actors responding to a wide array of incentives—all actors have a vital part to play in the pivoting of food systems toward, rather than away from, nutrition. These actions are different for each nation and for different areas within each country.” (HLPE, 2017: p. 119). The same applies for sustainability, yet rarely are the two considered together in policy actions. There is a need for research to identify the actions needed from production to consumption to enhance both the nutritional and environmental aspects of the food system. Actions that can incentivise the production and consumption of healthy foods while also reducing environmental impact and remove perverse disincentives to change are critical to identify and promote.

A group of foods that are both nutritious and environmentally sustainable are indigenous and traditional food crops (ITFCs) (Akinola et al., 2020). While ITFCs were once widely consumed, over the past half century, the world has increased its reliance on three major cereals-wheat, maize, and rice-to the detriment of diverse diets (Frison et al., 2006). Although there are about a dozen cereal crops that are used for food, wheat, maize and rice account for 94% of all cereal consumption (Ranum et al., 2014). In contrast, many crops of regional importance, including cereals such as sorghum, millets and rye, have lost their status (Khoury et al., 2014). Prior to colonization in sub-Saharan Africa, ITFCs like *Sorghum, Amaranthus* species, Bambara groundnut (*Vigna subterranea*) and other crops were the main source of food for communities, but there has been a post-colonial displacement of these foods as they are replaced by foreign staples like wheat, and a concomitant stigmatization of these ITFCs as “poor man’s food” (Demi, 2014; Chivenge et al., 2015; Mabhaudhi et al., 2018). As such, cultivation of ITFCs has become non-competitive and unattractive compared to the “major” crops that are promoted through formal seed systems and markets (Chivenge et al., 2015). The role of indigenous foods in diets continues to be eroded in line with global trends as Western diets and formalization of the food system continues apace (Drimie and Pereira, 2016; Mbhenyane, 2016).

However, the importance of ITFCs is being recognized. The African Model Law for the Protection of the Rights of Local Communities, Farmers and Breeders, and for the Regulation of Access To Biological Resources (OAU, 2001) emphasizes the contribution of indigenous farming systems in Africa to food sovereignty and security, and recognizes the need to maintain the genetic diversity, protected by Africa’s smallholder farmers, as critical for the continent’s economies, cultures, environment, food security and livelihoods (Munyi et al., 2012). In order to sustain these indigenous farming systems, there is a need to build markets and increase consumption: research suggests significant potential for innovation in indigenous plants to shift them onto people’s plates (Dlamini and Siwela, 2015; Mbhenyane, 2016). Yet there is limited innovation in policy and investment around these species (Pereira, 2017). Increasing their consumption and production will require strategic policy interventions and innovation in order to overcome dominant trends in the food system. This study aims to identify entry points in the food system that could, if effectively incentivized (and disincentives removed), enable greater production and consumption of a specific ITFC—sorghum (*S. bicolor*) in South Africa. Learnings from this particular case study can be relevant for other settings where the inclusion of ITFCs are sought. Sorghum plays an important role in food security in some of the poorest places around the world, and a recent review provides evidence that its production is influenced by some key factors including agricultural inputs, population growth/economic development and climate change (Mundia et al., 2019). One of the key areas for research identified in the review is the need to go beyond these broad trends and to look into more local dynamics for planning purposes. This case study offers just such a contribution to the literature, focussing on sorghum in the South African food system, but understanding that it sits within broader, global dynamics. As a nutritious alternative to the cereal staples currently consumed in South Africa, sorghum offers a lot of potential as it is ecologically sustainable with potential for innovation to allow it to be consumed in a wider variety of ways (Hadebe et al., 2017). It is already used to make several food products in South Africa, including malted meal, beer, fermented or unfermented porridge, stews (with whole grains) and bread, and (as immature sorghum) a fresh vegetable (Bichard et al., 2005), but with a stagnating market, interventions are required for it to reach its full potential.

## Methods

### Choice of Sorghum

Sorghum was selected for the study because it is one of the most important cereal grains for food consumption indigenous to the savannas of the African continent (de Wet, 1977).

There is archaeological evidence in the Sahara of the use of sorghum dating back 8,000 years (Wendorf et al., 1992) and that the domestication of sorghum likely took place in the Ethiopia-Sudan region in northeast Africa because the greatest plant diversity and variation in ecological habitats occurs there (OECD, 2016). Recent data from seed impressions on the Butana Group pottery from the fourth millennium BC in the southern Atbai region of the far eastern Sahelian Belt in Africa, show evidence for cultivation activities of sorghum (Winchell et al., 2018). It is clear that Sorghum formed an integral part of the caloric base of most Neolithic and Iron Age food-producing societies in sub-Saharan Africa (National Research Council, 1996) and following its domestication, humans moved cultivated sorghum across much of sub-Saharan Africa. However, sorghum is not restricted just to the African continent as many species are native to Australia and Southeast Asia (Lazarides et al., 1991). The cultivated sorghums have annual wild relatives native to Africa, Madagascar and the Mascarenes and introduced varieties as far afield as India, Australia and the Americas (OECD, 2016).

The species is highly variable with the division of cultivated sorghum into subspecies and races over the past century being somewhat archaic with many competing classifications, but there are officially 25 recognized species of sorghum, ranging from cultivated sorghum (*S. bicolor* subsp. *bicolor*) to its annual wild relatives (*S. bicolor* subsp. *verticilliflorum*) and annual weedy derivatives from hybridization between the two (*S. bicolor* subsp. *drummondii*) (OECD, 2016) (See the full nomenclature undertaken by Wiersema and Dahlberg, 2007). Sorghum has two main types-including a sweeter type (GM) and a more bitter type that contains more tannins (GH). It also comes in a range of colors-from white through to red. Sorghum’s adaptability to a range of environmental conditions has led to it being cultivated in substantially varied climates with two main belts of cultivation in Africa: (1) the northern belt from the Ivory Coast north to the Sahara, and east toward Sudan and Ethiopia and (2) The second African sorghum belt includes the races *Kafir, Bicolor*, and *Caudatum*, running north to south from Ethiopia to South Africa (OECD, 2016). Sorghum’s required annual rainfall ranges from 400 to 750 mm, which makes it an important crop for areas too dry for maize production and although it is primarily known for its drought resistance, cultivated sorghum can also withstand temporary water logging (OECD, 2016). Evidence from South Africa shows that different genotypes of sorghum have demonstrated adaptation to low water availability, emphasizing its drought tolerant capabilities (Hadebe et al., 2017). It is therefore ideal for production in water-scarce regions, especially under changing climate conditions such as those projected for South Africa, due to its high and stable water-use efficiency, drought and heat tolerance, and high germplasm variability (Hadebe et al., 2020a,b). Sorghum therefore holds much promise as a resilient option for farmers to plant under changing climatic conditions.

**Figure 1:**
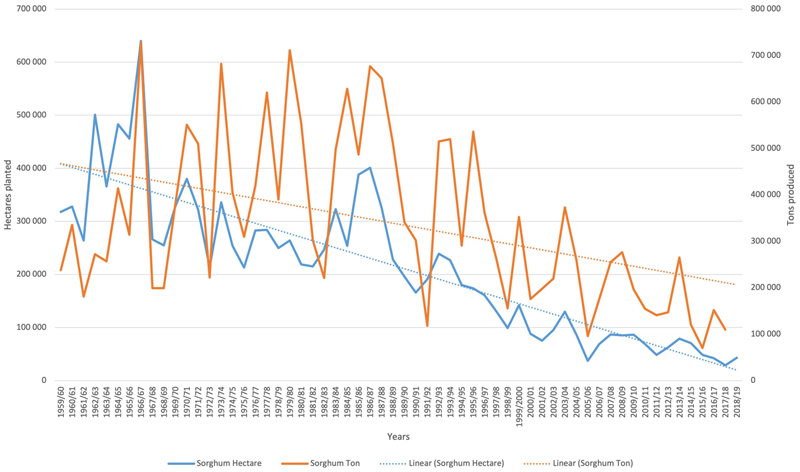
Area of sorghum planted (Ha) and amount produced (tons) in South African from 1959/60 to 2018. (Source data: GRAIN SA).

**Figure 2:**
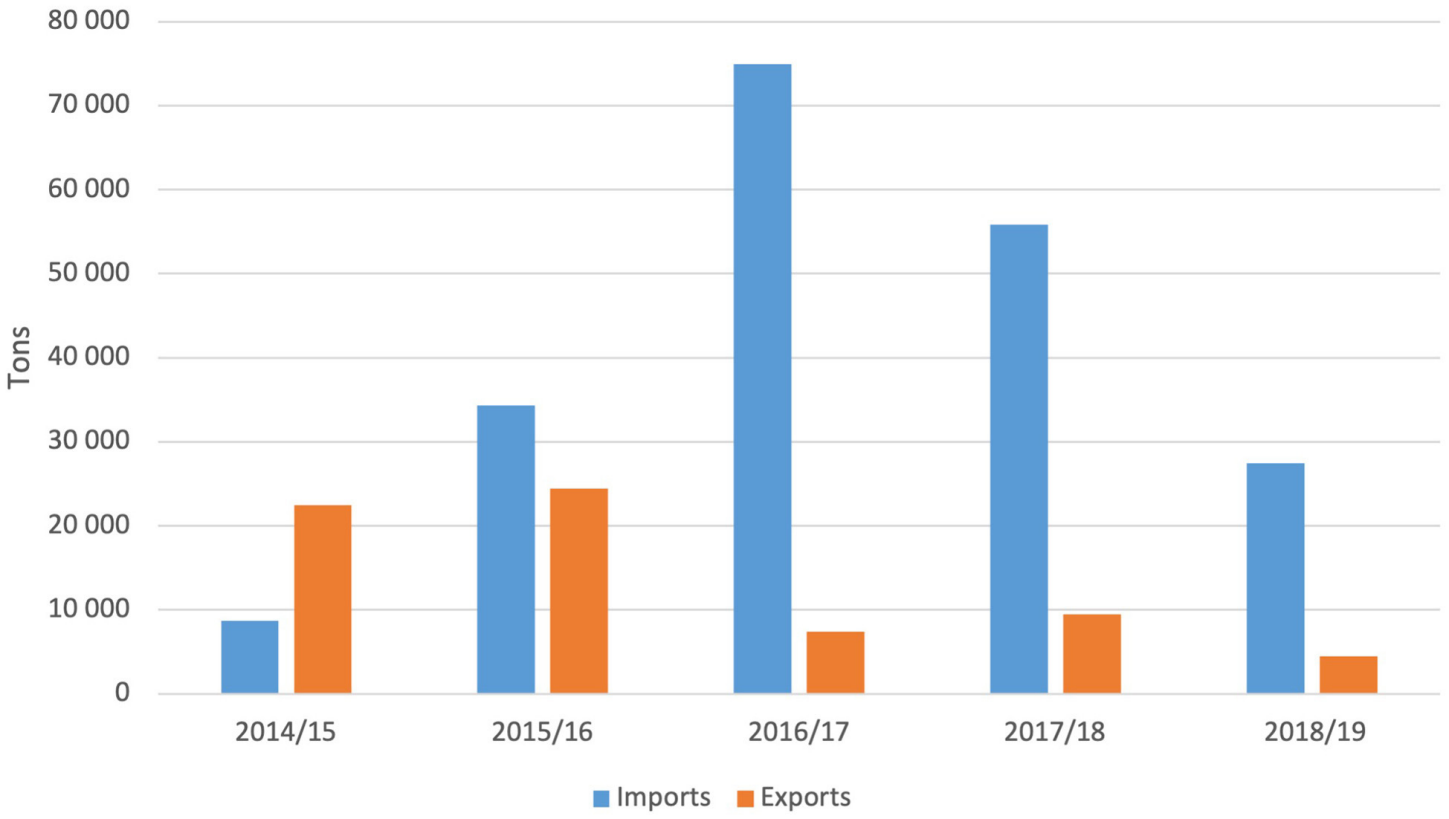
South African sorghum imports and exports 2014–2018 (Source: SAGIS).

**Figure 3:**
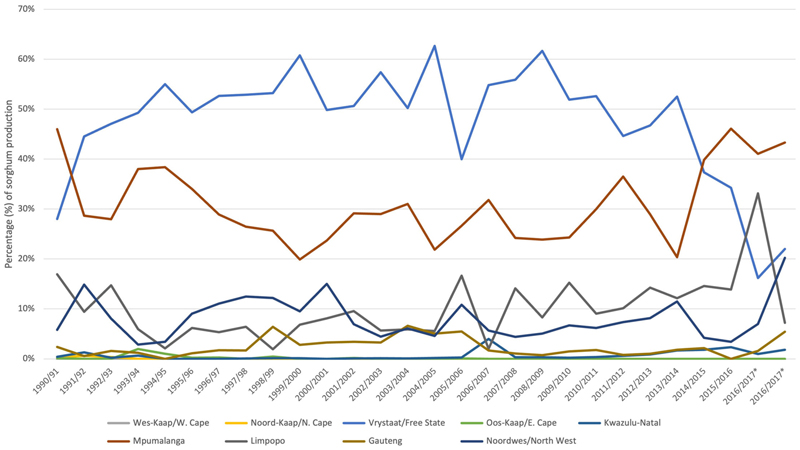
Percentage of sorghum production (tons) by province 1990–2017 (Source data: GRAIN SA).

Africa is the world regional leader in total production of sorghum at 25.6 million tons, but it has the average lowest yield per hectare at 967 kg ha-1 (OECD, 2016). In South Africa commercial production of sorghum has declined in recent years (Figure 1) with only 28, 800 hectares planted in 2017, producing 109, 855 tons of sorghum. South Africa has therefore started to import sorghum, mainly from the United States, but also from Botswana, Brazil, Lesotho, Malawi, Ukraine and Zimbabwe, whilst exporting some sorghum regionally: to Botswana, Chad, Namibia, Swaziland, Tanzania, and Zambia (see Figure 2). Most of the commercially grown sorghum in South Africa comes from the Free State Province, followed by Mpumalanga (Figure 3).

Nutritionally, sorghum is mainly carbohydrate, followed by protein, fat and fiber; it contains 1 percent less fat and has a variable protein content that is generally 1–2 percent higher than that of maize (National Research Council, 1996). However, it is deficient in some essential amino acids, most importantly lysine-it contains about 45 percent of the recommended lysine requirement (National Research Council, 1996). Sorghum has low protein digestibility due to its tannins—the higher the tannin content, the lower the digestibility- and so it must be properly processed, which is perhaps why sorghum in Africa is generally fermented. Sorghum has high antioxidant and anti-inflammatory properties that are attributed to these tannins and so there is a trade-off between protein digestibility and other health benefits (Awika et al., 2003, 2009; Awika and Rooney, 2004; de Morais Cardoso et al., 2017).

### Study Methods

In order to draw on the richness of individual narratives and experiences, this paper employs an adapted “follow the thing” approach from the discipline of human geography (see Cook, 2004, 2017; Cook and Harrison, 2007; Pereira, 2010). The “follow the thing” method traces encounters with the material subject (sorghum) through different starting points in the value chain to unpack system complexities and potential interventions. The approach has similarities with other methods focused on tracing the product through the system (Hawkes and Ruel, 2020), such as value chain analysis or commodity chain approaches that focus on embedded power relations between production and consumption (Hartwick, 1998; Raikes et al., 2000; Gereffi et al., 2005).

Many research approaches tend to see food systems as linear. Yet food systems are complex adaptive social-ecological systems (Ericksen, 2008) making it important to employ a research methodology that can capture this complexity in a way that provides practical insights whilst not falling into a trap of assuming linear relationships. The “follow the thing” method enables an understanding of the connection to the more personal social-cultural drivers of change in the food system, such as cultural preference, aspirations and even the narratives that drive decision-making around food by tracing stories of particularly poignant individual encounters with the subject matter. This is necessary to provide a more grounded picture of reality as it plays out in the lived experiences of people, not just abstract value chains that move products from point a to point b (Pereira, 2021). This methodological approach allows the researcher to unpack the complexity of the food system through encounters with a specific subject and the personal stories associated with it. It uses an inductive approach to elucidate challenges and opportunities in the sorghum value chain based on people’s actual experiences of the product (Cook, 2004). It does not aim to provide an in-depth analysis at each stage of the value chain, for which many more interviews would need to be undertaken at each point, and is therefore limited in the depth of information that it can provide. Rather, it attempts to provide a more holistic overview of a particular commodity in a system from a range of individual perspectives and through this perhaps to offer some novel insight that might be missed at a more granular level. There will be gaps due to the personal encounter aspect of the method that cannot encapsulate the full complexity of a food system, and it is likely that another person replicating the process would have different moments of encounter and therefore a different narrative to convey. However, this subjectivity, when acknowledged, should not limit the learnings that can be garnered from such an undertaking as it’s often in the personal encounter that insights are revealed (Pereira, 2021).

The follow the thing method uses both standard qualitative interview techniques, as well as photography and experience as data. Qualitative information was primarily obtained through 13 semi-structured key informant interviews that took place between the last quarter of 2018 and the first quarter of 2019. The interviews started from the researcher’s own networks who could talk to the different encounters of sorghum and then snowballed to ensure each core aspect of the value chain had been captured in at least one interview (see Table 1 for a list of key informants).

Each interview had a core set of themes for discussion:

1. Constraints to production/ marketing/ consumption
2. Current market and future opportunities
3. Current government policies and programmes
4. Suggested government interventions to improve the production and consumption of sorghum
5. Other potential interventions for overcoming constraints and opening up opportunities

The interviews were recorded either on tape or in writing and then analyzed for responses based on answers to the five core themes. As emphasized above, the data are not meant to be representative of the entire sorghum value chain, but to present stories that are indicative of experiences across the food system that touch on different aspects of sorghum as a material that is produced and consumed across the country in different ways. From these stories and especially in their intersections, a picture of what interventions are deemed more appropriate from a range of perspectives emerged from the analysis. That said, throughout the paper, the information from the interviews is triangulated either through primary data from national databases (including GRAIN SA and the South African Grain Information Service-SAGIS) or through secondary sources, both in the peer-reviewed and gray literature, including Masters theses and publicly available government documents, like the minutes of Sorghum Forum meetings. The storylines of the encounters are more fully described in Pereira (2021), whereas in this paper, there is a greater reliance in the results on the gray literature and statistics to bolster the recommendations suggested for policy.

Ethical clearance was granted by City University of London, reference number Soc-REC / 80025567 / 22-04-18 and all whose names are used gave their permission otherwise where there is an asterisk next to a name in the paper, this is a pseudonym.

## Results and Discussion

The process of encountering and talking with people intimately involved in South Africa’s sorghum system led to the identification of five key entry points where creating incentives and removing disincentives have potential to enable greater production of sorghum in South Africa and thus contribute to improved nutrition and environmental sustainability in the country. Following the typology by Downs et al. (2020), these largely map on the availability, affordability and appeal of sorghum as a healthier and more sustainable food product. Whilst the richer narrative encounters are presented in Pereira (2021), the results are presented here with data from secondary sources to back up the claims made by interviewees and then directly discussed with reference to the literature.

### Availability: Agricultural Research

The South African Agricultural Research Council (ARC)’s hub for sorghum is at the Grain Crops Institute in Potchefstroom. Sorghum is one of the ARC’s mandate summer grain crops and although it has been researched since the 1980s, research capacity and funding has been very small compared to other cereal crops like maize and wheat. Historically, the ARC-Grain Crops breeding priority was improvement for bitter sorghum varieties, grown and malted for use in beer, but as people’s preferences have changed toward clear beer (not made from sorghum) and increased awareness of the health benefits of sorghum, emphasis has moved to sweet sorghum (no or low tannin types) that can be milled for consumption as food and can also be used for ethanol production (Nemera). According to Nemera, the ARC’s chief sorghum researcher, over the years the ARC has made sorghum germplasm collections sourced both locally and abroad, and at present maintains over 3, 800 accessions. Most of the accessions were assessed in batches for genetic diversity using morphological and nutritional traits. So far, sorghum has received little resource allocation for research and development compared to maize, but efforts have started to make the research approach more interdisciplinary, involving breeders, molecular biologists, agronomists, crop protection scientists and food technologists/nutritionists.

There is great potential for isolating potentially useful genes in South Africa because sorghum is an indigenous crop and there are races that have diversified there (Nemera). Smallholders still grow traditional varieties, but research support has been very low (Lawrence). The Limpopo province, where sorghum is mainly produced by smallholder farmers in intercropping systems, faces a major challenge in improving production and productivity (Temba). According to Nemera, in addition to varietal improvement and enhanced crop management, the use of quality seed significantly contributes to improved productivity of sorghum-one of the main challenges to its production. Open-pollinated varieties (OPVs), rather than hybrids, are the main varieties used in Limpopo and by other smallholder farmers. In 2010, the ARC-Grain Crops Institute started a sorghum seed production project with a group of smallholder farmers in Limpopo province with funding obtained from the then Department of Agriculture, Forestry and Fisheries (DAFF). Twelve farmers from the Difahlane project, in Makhuduthamaga municipality, and four farmers from the Ka-Dikweneng project in Lepelle-Nkumpi municipality, produced certified sorghum seed with assistance by the ARC and local extension officers. The standards for seed production have been met and enforced by the South African National Seed Organization (SANSOR), which controls the seed certification scheme. This success story of how research with farmers can translate into real change for small-scale farmers indicates what the potential is for more such projects to be undertaken. According to the Agricultural Research Council, training farmers in community-based seed production can have an impact on farmers’ access to seed, provided that seed production costs can be kept lower than those of the formal seed sector and that the quality of the seed produced meets the farmers’ expectations.

In the private sector, Pannar is the main seed company that worked on hybrid sorghum seeds, but since it was bought by Pioneer, most of its breeding work on sorghum has stopped and farmers are having to rely on imported seeds (Mark*). Pannar no longer maintains a specific breeder in South Africa and instead focuses on research overseas, mainly in the USA. Locally, there is a demand for hybrid seeds that are adapted to local conditions and some small seed companies are wanting to work with the ARC to get more capacity (staff, facilities, collaborators, testing sites). One of the main drivers of this work is climate change because sorghum is highly adaptive to heat and drought stress (Rosenow et al., 1983; Hadebe et al., 2017); conditions that are likely to become more prevalent across the African continent (James and Washington, 2013). Commercial farmers also want hybrids as they believe that these have a higher yield (Nemera). According to the interviews, most farmers would be keen to work with researchers on testing improved varieties as they are keen to plant sorghum as part of a crop rotation system. “*Getting improved, higher-yielding varieties of sweet sorghum, and focussing on local varieties not imported ones, will encourage farmers to grow more”* (Nemera). Furthermore, sweet sorghum types can not only be used for food, but also for bioethanol as they are more digestible and have a higher starch content. The strong international research on maize following the Green Revolution (GMOs, hybrid varieties etc.) has resulted in maize yields increasing to 3–5 tons per hectare in South Africa, although many of the characteristics that are bred are not those most desired by smallholder farmers (Fischer, 2022). However, sorghum yields have remained stagnant, so that now it makes up only about 2% of total grain production in South Africa. A strong focus on investing in appropriate sorghum research is needed to make it competitive with maize, and even if yields are never equal, other benefits such as drought-tolerance could be promoted.

Allocating such funds could allow farmers to be more competitive when growing sorghum. The relatively low yields need to be addressed through increased research on hybrids and OPVs that are developed in conjunction with commercial and small-scale farmers. More money therefore needs to be invested in research to improve varieties. According to Mark*, despite a renewed emphasis on indigenous crop research in the rest of Africa, South Africa does not seem to attract partnerships with research institutions to pursue breeding programmes. A concerted move to attract international funding needs to happen in conjunction with improved national coordination of funds.

Under the apartheid regime, the agricultural research system focused on commercial production, but this focus reoriented after 1994 when the smallholder community of mainly black farmers became a core focus of attention. Despite the political reorientation, South Africa’s agricultural system remains dualistic with around 35, 000 (largely white) commercial farmers producing almost all of South Africa’s agricultural output with about 4 million (mainly black) small-scale farmers contributing very little (Aliber and Hart, 2009; Pereira and Drimie, 2016). Addressing these very different concerns is a difficult, yet not impossible task for agricultural research. The ARC gets their funding through Department of Agriculture Forestry and Fisheries (now the Department of Agriculture, Land Reform and Rural Development), but this is usually only sufficient to pay for salaries, infrastructure etc. and not for new research projects. However, at the provincial level, there is money allocated to agriculture from the Treasury, but they do not necessarily have the capacity (human and infrastructural) to undertake this research (Nemera). Combining the different funds, by making use of the resource capacity of the ARC and combining it with the money at provincial level for research done in conjunction with extension officers and farmers, could have a real impact. Meaningful co-produced research, especially with smallholder farmers is critical and could make these farmers more competitive. Research has shown that farmer-led seed systems have the capacity to supply seeds of good quality and it is important to recognize such systems and promote them as a means to meet the ever-evolving needs of smallholder farmers across the continent (Kusena et al., 2017). However, the research problems that need to be addressed extend beyond the agronomic to include grain marketing and processing as well as food product development, marketing and consumption. This is where clear partnering with universities and other research institutes, and funding these collaborations, is critical.

### Affordability: Markets

Internationally, the sorghum trade has been declining since the 1980s, with the top five exporting countries (USA, Australia, Ukraine and France) selling primarily to meet demand for livestock feed (Pingali et al., 2021). Sorghum has to compete with maize as a preferred grain for feed and is only really competitive when its price is below that of maize (Pingali et al., 2021). This is a problem sorghum faces in South Africa too, but as a food crop rather than as feed. The local South African market for sorghum is ~260 000 tons, of which in normal years half is produced locally and the rest is imported. Of what is produced locally, some goes toward industry, for wet beer brewing (~6% of total production) and commercial use. Feed is a small component now (~5,000 tons per year-2% of the total) as it cannot be used in the formal feed sector due to the high tannin content of the bitter variety and erratic supply (*Bob). According to *Bob, feed suppliers have a set recipe for their feed that they cannot change just because there is an excess production of sorghum 1 year and not the next. He argues that there could be a good market for feed if sweet varieties were grown and sold at 80% price of maize, but currently no producer can meet these requirements. Bioethanol has been explored as a potential market for sorghum, but many of the projects in the Eastern Cape have not come to fruition although this could be an important component of the market in the future (Makanda et al., 2011). The main consumption of sorghum in South Africa is therefore for human consumption.

South Africa’s has a series of “Grain Trusts” (Maize Trust, Sorghum Trust, Winter Grain Trust, Oil & Protein seeds Development Trust), which were formed following the closure of the agricultural marketing boards in the early 1990s. The main purpose of the Trusts is to support their respective industry, especially with regards to information and in particular to undertake and/or financially support scientific, technical or industrial research. This contrasts with the agricultural marketing boards that directly intervened in the marketing of produce. Under the previous board system, farmers could produce sorghum profitably as the boards would ensure that all sorghum was purchased. “The dismantling of the control boards with the enormous development capability they had exercised in establishing white agriculture, and the suspension of their protective and beneficial schemes; left the South African agricultural industry in general and the emerging sector in particular more exposed to the ravages of the market than in any comparable economy in the world” (Government of South Africa, 2020). Now, while the Trusts inherited the remaining assets of their respective boards, they no longer enable secure markets. The only remaining protection for sorghum is a 3% tariff on sorghum imports from all regions except the EU and SADC (SAGIS, 2020).

Maize is sorghum’s direct competitor and it not only has higher yields, but maizemeal (the main food product of maize in South Africa) is also a zero-rated foodstuff, i.e., it is exempt from value-added tax (VAT). As maize yields have improved relative to sorghum over the past few decades, due to a much high investment in research and development globally, with prices being equal, farmers get more per acre for planting maize than for sorghum. The lower investment is also directly related to research capacity, as pointed out in the previous section, that is correlated with the higher financial capacity of the Maize Trust relative to the Sorghum Trust to invest in research and innovation. As the trusts rely on industry money, with the maize industry maintaining its hold and the sorghum industry becoming less competitive, this has also meant less funds available to the Sorghum Trust and therefore less funds to go into research for improved productivity resulting in a positive feedback loop. With a decline in production over the past few decades, sorghum is now seen as a niche crop, not a staple commodity. Mark* informed me that up until a few years ago, one of the biggest corporate buyers and processors of sorghum in South would actively target a specific group of farmers and offer them contracts at a premium price in order to keep them growing. However, as it became increasingly too expensive to compete with maize at this high price without a VAT exemption for their sorghum products, this contract is no longer offered and so these farmers are no longer planting. The decline in sorghum production has meant that, for buyers, domestic sorghum has traded at a high price for the past 10 years or so, but this has still not been a sufficient incentive for farmers to grow sorghum instead of maize, due to productivity differences. This has made it difficult to source high quality sweet sorghum for food products in the country. It has also made it next to impossible to offer a premium price to small-holder farmers as an incentive for them to enter the market.

Due to this decrease in production, South Africa has had to import sorghum in order to meet demand. Even though these days South Africa’s processors rely on imported sorghum, some “*would love to buy locally*… *as we’re a pip in the world supply*” and “*it’s painful to buy internationally and try to get it here*” (*Sue). Most sorghum produced internationally is used for feed, but for food products in South Africa, companies require a certain quality and specific grade. Once the correct product has been sourced, it can still be difficult to divert the relatively small amount of sorghum that South Africa requires away from much larger international shipments; “*if you’re not there, and it’s not a waxed system, getting it here is really difficult*” (Sue). South Africa imports much of its sweet sorghum from the Americas rather than the Ukraine and India, relying principally on the USA because the logistics from Brazil and Argentina can be difficult (*Sue).

**Figure 4:**
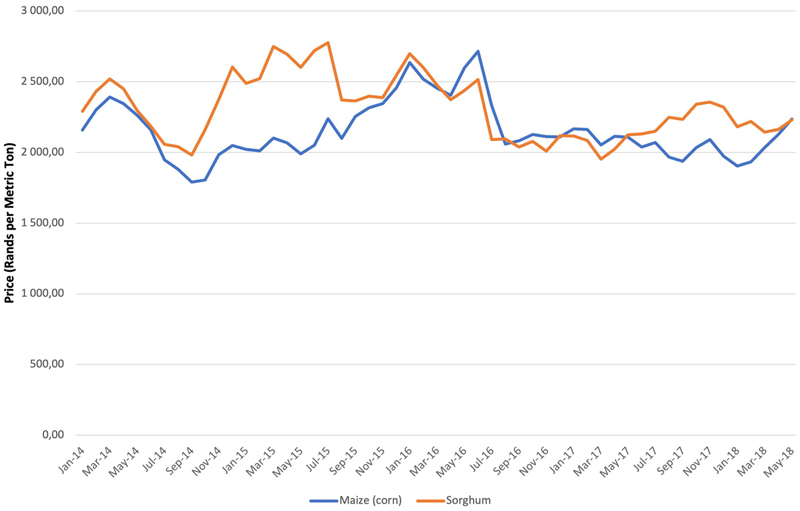
Price of maize and sorghum January 2014 to May 2018 in ZAR per ton. Source: https://www.indexmundi.com.

As South Africa relies on the international market for its sorghum, and since import parity drives price, sorghum is generally either comparable to or slightly more expensive than maize on the international market (Figure 4). However, when it comes to the price that consumers pay, given the reduced cost of maize due to the VAT exemption, sorghum becomes uncompetitive. This makes maizemeal a much more price competitive product than sorghum and in the shops, “*whilst it is possible to pay in excess of 10.00 ZAR per kilo for sorghum, it is possible to get maizemeal at around 5.00 ZAR per kilogram*”^1^ (*Mark).

Internationally, sorghum like other coarse cereals other than maize, has been neglected with support policies favoring the production and consumption rice, wheat and maize (Pingali et al., 2021). Similar to the VAT exemption that encourages maize consumption in South Africa, favorable procurement prices and subsidies to rice and wheat in Asia have led to a decrease in the consumption of sorghum. In India changing food preferences due to rising income and growing urbanization are further leading to a substitution away from coarse grains like sorghum toward fine cereals (Gali and Rao, 2012). Without a concerted effort to reinvigorate these resilient grains through innovation and policy support, these declining trends will continue. Unlike in many other developing countries and especially in Africa where it is usually grown for domestic consumption and stored in small quantities, with only small surpluses make its way to the markets (Pingali et al., 2021), there is commercial production of sorghum in South Africa meaning that that there is potential for the domestic market to overcome the traditional challenge of low and variable volumes, high transaction costs and long distances to larger markets. However, affordability is a critical component of developing a thriving sorghum market in South Africa.

### Affordability: Trade and Taxes

Sorghum prices are now directly linked to SAFEX (South African Futures Exchange) and pre-planting contracts are still afforded, but according to an international price. The sorghum contract on SAFEX can play a valuable role to ensure market and price transparency, but is not used to its full potential by market players. One of the possible reasons is because the previous size of the contract (100 tons) exceeded the entire production of a small sorghum producer. A smaller contract size on SAFEX may increase the use of the exchange for trading in sorghum as is the case for soybean, where the contract size on SAFEX is 50 tons and it is far better supported than the sorghum contract (Bob). Therefore, on 2 November 2018, the Sorghum Forum resolved that a formal request will be made to the Johannesburg Stock Exchange Commodities Exchange to consider reducing the size of the SAFEX sorghum contract to 30 tons. As of 2020, this has been accepted (JSE, 2020) and over time might play an important role in incentivising smaller producers to enter the market by getting a guaranteed price through this mechanism.

If South Africa were to significantly increase local sorghum production, developing an export market for sorghum shows potential to be lucrative because it means that if there is over-production in 1 year, there is a mechanism to get rid of the surplus if it cannot be used for feed. South Africa used to export sorghum to Botswana, but they have now become almost self-sufficient, allegedly because farmers there are getting a premium price for their product (Bob*). China is a big export market for Australia where they brew a sorghum-based alcohol, Kaoliang, that is apparently the most popular in the world.^2^ Accessing international markets could be beneficial for South Africa, but it would require a lot of research and most likely the establishment of bilateral trade agreements at a national level.

According to the Sorghum Forum, one of the major constraints facing the industry is the fact that sorghum is subject to Value Added Tax (VAT), which is not the case with maize and most wheat products^3^. Sorghum therefore has a 15% disadvantage to its competitor grains, which can historically be linked to its utilization in beer. Applications have been made to Government to exempt sorghum from VAT, but this has not been successful thus far. The opportunity of zero rating the VAT on sorghum was a strong incentive that emerged from many of the discussions. The argument is that if sorghum can be bought at the same price as maize, then people will start to shift their consumption because of its health benefits and because its indigenous heritage has marketing potential. However, there is some doubt as to whether lowering the cost would really be an influencer to get people to eat it more. Some think that there rather needs to be a concerted effort made in shifting perceptions about certain foods that are not seen as aspirational for historical reasons. It seems that any financial intervention to lower the price of sorghum would need to go hand in hand with a strong awareness raising campaign.

### Appeal: Innovating Novel Products

**Figure 5:**
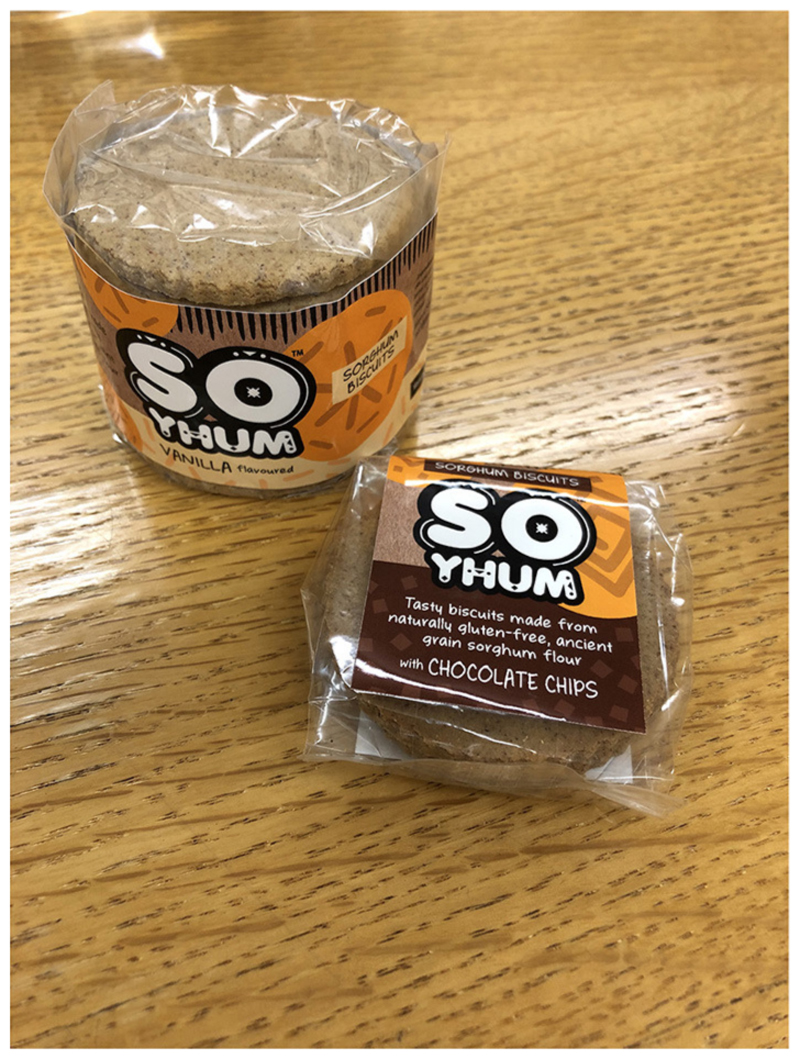
Image of So Yhum! Sorghum biscuit products produced by researchers at the University of Pretoria.

According to Riette, sorghum has an interesting taste profile. As opposed to maize, which is bland and therefore allows the consumer to eat lot of it without getting tired of the flavor, sorghum has a stronger taste. Riette says she likes to follow rye research and product development because it is a grain that also has a strong flavor, but many people appreciate this complexity and actively seek it out. She argues that consumers should be pushing themselves to appreciate more complex flavors. “*It is also necessary to describe and profile sorghum’s taste profile as ‘bitter’ and ‘sweet’ doesn’t really say much*” (Riette). Together with colleagues at the University of Pretoria, she has been developing processed sorghum products for the growing numbers of consumers in urban areas looking for affordable, convenient food. One such product is So Yhum! biscuits (see Figure 5). Similar interventions are underway in countries like India where the research councils and institutes are working on processing sorghum into a variety of products, and marketing them through Heritage Fresh retail outlets and Choupal Fresh (ITC) and other unorganized retail stores in Hyderabad (Pingali et al., 2021). Sharing across these developing country contexts could prove extremely fruitful as lessons are learnt-like the failure of cassava bread to take off in Nigeria despite decades of innovation and government support- and challenges are overcome. In the case of cassava in Nigeria, whilst decades of investment had gone into enabling the production of cassava bread in order to create markets for cassava farmers and reduce billions of Naira spent on wheat imports, once the products hit the shelves thanks to government incentive schemes, they were not taken up by the public as insufficient market research had been done as to why people might prefer wheat bread or the stigmas associated with cassava that limits its potential to be an aspirational food (Pereira, 2018). One of these challenges for the South African market has been the strong flavor of sorghum compared to maize, but also a potential for lauding its health benefits.

Phenolic compounds in sorghum are responsible for its stronger flavors. Sorghum has some unique compounds not found in other cereals and these could have potential health promoting, anti-cancer, and anti-diabetic properties (Awika and Rooney, 2004; Awika et al., 2009; Park et al., 2012; de Morais Cardoso et al., 2017). Sorghum’s bitterness comes from its tannins. For food purposes, the bitter (GH) type is usually not used, but, these varieties are more bird resistant. “*It’s not that people cannot get used to more bitter flavors-some foods like tea and beer are bitter and that’s what makes them attractive*” (Riette). However, there are other, more negative health implications associated with tannin-containing varieties. The compounds that cause bitterness also bind to proteins and divalent metal ions and when they bind the nutrients, they cannot be absorbed. This means that the food has low protein digestibility and poor mineral absorption, which is a negative in South Africa where there is a problem of anemia in the population.^4^ On the other hand, the bitter types have other health promoting properties through anti-oxidants, with high potential for being health-promoting ingredients (Awika et al., 2009). Innovation to add other compounds like cowpea, roasted coffee, wheat flour and barley to sorghum products have all shown consistent results in improving the nutritive value, antioxidant properties and phenolic compounds (Salazar-López et al., 2018). Different ways of preparing sorghum can have different impacts on its nutritional properties, for example fermentation and extrusion cooking can have various benefits (Duodu et al., 1999; Cardoso et al., 2014; Salazar-López et al., 2018). Studies on chickpeas, sorghum, green gram and wheat showed that in general, sprouting and roasting provided more bio-accessible polyphenols and that there is an increase in tannin content of both the cereals on sprouting as well as roasting (Hithamani and Srinivasan, 2014). Furthermore, pre-cooking sorghum can be more convenient for consumers and can also give it interesting textures. “*I think it’s open to much more investigation in the culinary sense*” (Loubie).

Yet as a food, sorghum also faces marginalization since maize became the main staple of the country. “*People think that maize ‘pap’ is traditional, but it isn’t and we’re also not eating it the right way by not nixtimilising it as maize was originally prepared in Americas* (See Moreno-Rivas et al., 2014). *We have the solution, but we don’t use it. Apartheid government promoted maize for politics-it was seen as a waste food, but the new government hasn’t changed this at all*… *Changing mindsets is difficult*… *(we need to) promote it (sorghum) as a healthy, indigenous grain”* (Mpho). Unfortunately, we now have a narrow idea of how to use sorghum and have been accustomed to maize, using sorghum only for ancestral offerings… “*you can’t communicate with the ancestors with maize because they don’t understand what it is and they get confused*” (Mpho).

Table 2 lists some of the main sorghum products and brands associated with the mainstream commercial market (i.e., not health stores or specialty food shops). As sorghum products are not the main brand for many of these food processing companies, they are often starved of marketing and attention, with more focus going onto other, more lucrative products (Sue*).

Interestingly, even when being served in the best of high-end restaurants, Wolfgat in Paternoster, sorghum porridge still comes with a stigma.

Roelie: “*One guest complained that it was famine food: How dare you feed me porridge in a high-end restaurant? And she sent it back*…”

Kobus: *“*… *that was the reaction she had-how dare you serve me poor man’s African food? It’s supposed to be a high-end experience*… *no, it’s funny, some people just (have that reaction)*…

**Figure 6:**
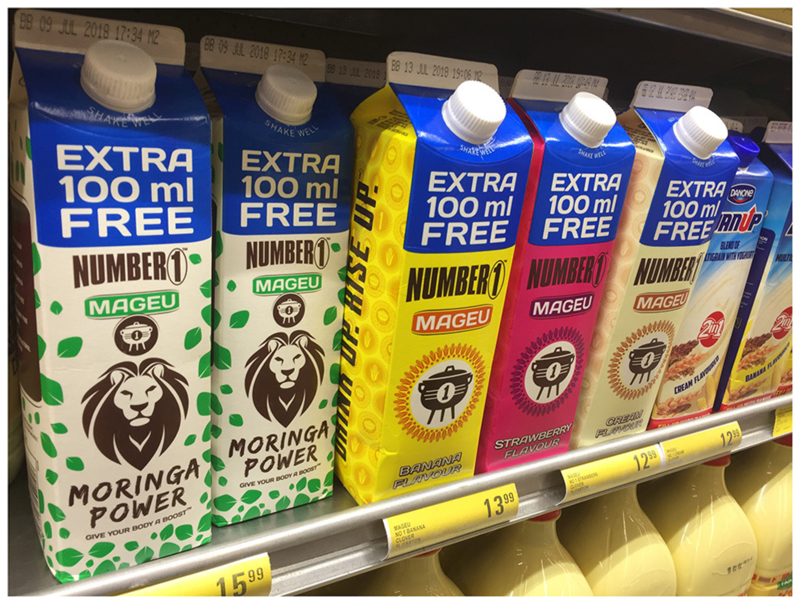
Images of Mageu in a variety of flavors.

There has been a recent resurgence in popularity of “indigenous ways of cooking” like fermentation. If you go to supermarkets, mageu^5^ has increased in consumption even in mainstream, and more upmarket areas. It seems that there is a trend to add some indigenous foods to products, like moringa (Figure 6). “*There appears to be a shift in local consumers mind that we’re more interested in indigenous. I think there is a big market space and gap for proudly South African, proudly African, but you need interesting products and products that people can associate with. So mageu and some of those products that are linked to yogurt and similar packaging that makes them more marketable*… *“Super food” and “Ancient grain” branding* (can also be helpful)” (Riette).

It seems that a focus on both sorghum’s health benefits as well as its status as an indigenous food could be leveraged to encourage an increase in consumption if there is sufficient availability and development of innovative products. This means not just focussing on high-end consumers, but also to look at how taste palettes can become as accustomed to sorghum as they are now to maize products.

### Appeal: Incentivizing Demand

Raising awareness about the benefits of sorghum as a viable alternative grain was perceived by all interviewees as really important. Sorghum is an extremely versatile grain-you can make sourdough bread, flapjacks, baked goods and many more things from it if you are willing to experiment. Mpho reminisced about Mosoko buns that were part of school feeding programmes under apartheid. She says that sorghum flapjacks remind her of those and recommends current school feeding programmes to give kids sorghum flapjacks. “*You can’t feed kids the same thing every day, sorghum is versatile enough to offer something new all the time*” (Mpho). Incorporating sorghum and other indigenous grains at school level could be an important intervention not just in terms of nutrition, but also as a way to incorporate knowledge of these foods from an early age. For example, a study incorporating African indigenous leafy vegetables among school-going children in Kenya showed these to be an important intervention against malnutrition (Wakhanu et al., 2020).

The potential of introducing sorghum in schools as part of a school feeding process could have multiple benefits-both as a steady market for small-scale farmers and as a healthy meal for children. As suggested by Malebogo, incentivizing the planting of sorghum in school gardens could also go a long way in raising awareness about the benefits of the crop and in incorporating it into diets. Finally, an active campaign to communicate the health benefits of sorghum as part of a nutritional diet through healthcare workers and in school communities could be beneficial in raising people’s understanding about the crop and countering existing stigmas.

All of the food innovators interviewed are experimenting with different ways to cook sorghum to make it more nutritious, delicious and convenient. For example, Kobus van der Merwe of Wolfgat is using sorghum as an alternative grain for gluten-free bread and also as a porridge to be served as dessert (Pereira, 2021). There are also nutritional benefits of certain cooking techniques; by letting it soak and sprout, the protein content of sorghum goes up (this is also the beginning of the malting process). The digestibility of chickpeas changes once they’ve sprouted, and this is often used by people on a raw food diet to get increased protein. The work by the University of Pretoria and their new impetus to market the products that they have developed could further help to share the benefits of sorghum and to push research further in understanding how to maximize the nutritional benefits of this grain. Having it on menus in restaurants, thereby making it accessible to ordinary South Africans, is also important- and not only in high-end establishments, but in ordinary cafés too (Pereira, 2021).

Some believe that there are definitely opportunities to expand the human food market, of sorghum but that it will always remain a niche product and so this expansion is relatively limited. They acknowledge that sorghum’s excellent health properties have driven a small increase in demand for sorghum by health-conscious consumers. Others think that there is a much larger potential market if there is increased awareness about sorghum and its benefits. Malebogo suggested that a public health drive aimed at combating non-communicable diseases could highlight sorghum as an affordable African superfood that is an important part of a healthy diet. “*If sorghum were marketed as an indigenous wholegrain, I imagine it could gain popularity given its relatively low cost and health benefits*” (Malebogo). A greater awareness of the diets of different ethnic groupings within South Africa could also improve visibility and uptake.

## Conclusion

The South African government has identified sorghum as crop of interest and in 2019 the Department of Science and Technology planned to conduct an impact study on sorghum, the outcome of this study should highlight the major issues that hamper the sorghum market. Hopefully the outcomes of this study will lead to better government understanding and support of the industry. What is clear from following sorghum in the South African food system is that it is an indigenous food with a rich and complex history. It has a comparable nutrient value to maize whilst also having high antioxidant and anti-inflammatory properties and when consumed as a whole grain helps meet requirements for dietary fiber. At the same time, it is more adaptive to climate variability, showing tolerance to drought conditions. It thus has the potential to help build resilience in the South African food system. However, there is a clear need to align the recent innovations around sorghum that has been happening amongst private individuals like chefs with the broader sorghum value chain that is floundering. Coordination between stakeholders is key and the current mechanisms do not seem to be functioning adequately to hold the relevant knowledge exchange. There is also a fundamental need to learn from what is happening within other country contexts, like India, where there is also a drive to support increased production and consumption of these more resilient and nutritious grains. (Pingali et al., 2021), set out a variety of policies in sorghum producing countries that could be considered in the South African context. For example, the Nigerian government’s attempt to set a guaranteed minimum price failed due to lack of funding and logistic constraints, but mechanisms like SAFEX or guarantees from large processing companies like Tiger Brands could overcome such constraints. In the longer-term, the potential for a sorghum food market to have to compete with livestock feed and ethanol production is a concern that needs to be considered in current policies.

A clear message from this research is that this may be a very opportune time for stakeholders interested in more diversified, healthier food systems to get government policy changes and significant private and public sector investments into sorghum research and value chain development. South Africa’s industrial sector has sufficient technical capacity to produce a diverse range of sorghum-based products, but ensuring that these are affordable and that there is sufficient production is not an easy challenge to address. However, there are clear areas where policy interventions could bolster the sorghum value chain. These include zero-rating VAT on sorghum products, investing more extensively in research and marketing and coordinating better across diverse stakeholders, raising awareness about the health benefits of sorghum and using public procurement as a way of instigating a market for novel sorghum products.

These are clear policy interventions that could bolster the sorghum value chain in South Africa whilst improving smallholder farmers’ livelihoods, making a nutritional food more accessible to South Africans and developing a local market for a crop that is resilient to projected climatic changes. Compared to maize, which is a staple food in South Africa, sorghum is relatively apolitical and is not as threatening to vested interests (Bernstein, 1996). It could therefore be used as a mechanism to engage multiple stakeholders (small and large-scale producers, millers, large and small industry players, government, civil society) about what a more sustainable and healthy food system in South Africa could look like, and what interventions are needed to get there.

If South Africa were to get it right in terms of developing a legitimate local and potentially even export market for sorghum, it could be a critical case study from which other countries facing similar concerns, like Nigeria, Ethiopia and India, could learn. How to engage an innovation system around indigenous crops that acknowledges indigenous knowledge systems and then to link it to address existing challenges and opportunities within the broader food system is a globally recognized problem (IPES-Food, 2016). Understanding the case of sorghum in South Africa could be a first step toward wider appreciation of and investment in this area of study for the innovation and policy communities.

## Figures and Tables

**Table 1:** List of key informants and the stakeholder group they represent.

Name/pseudonym	Stakeholder group
Malebogo Ngoepe	Urban Consumer
Sue*	Buyer and processor
Nemera Shargie	Research
Bob*	Industry and commercial farmers
Mark*	Industry and investment
John*	Industry
Kobus van der Merwe	Chef and innovator
Roelie van Heerden	Consumer and Innovator
Loubie Rusch	Indigenous food innovator
Mpho Tshukudu	Dietician
Riette de Kock	Research and innovation
Lawrence Makhapili	Civil society and smallholder farmer in Kwa-Zulu Natal Province
Temba Chauke	Smallholder farmer in Limpopo Province

* *refers to a pseudonym*.

**Table 2:** Table of commercial sorghum products for human consumption in the mainstream market (based on data from SAGIS and Grain SA).

Type of sorghum product	Percentage of total production used	Main companies
Malted sorghum (e.g., Maltabella porridge, King Korn malted sorghum, Quick brew original beer powder)	~ 36%	Tiger Brands, Nkosi Foods, Danhauser Malt
Meal/cereal (Mabele cereal, Morvite, sorghum flour to use in biscuits etc.)	~54%	Tiger Brands, Nola (owned by Rainbow), Afgri, Brenner Mills, Botshelo Milling, Progress Milling, Pioneer Foods
Non-human consumption (animal feed, ethanol etc.)	~10%	

## Data Availability

The original contributions presented in the study are included in the article further inquiries can be directed to the corresponding author/s.

## Footnotes

1 As an indication of this rough comparison, the cheapest Own Brand mabele meal product in the retailer Pick ‘n Pay’s online store is ZAR26.99 for 2kg whereas White Star super maizemeal is ZAR22.49 for 2.5kg.

2
https://www.theatlantic.com/health/archive/2009/04/is-this-the-best-selling-liquor-in-the-world/13060/

3 According to Kobus, there is a precedent for subsidizing local foods. The all-but-forgotten indigenous bean heerenbone (no English translation) were subsidized in the 1930s and the 40s.

4 Overall in South Africa, the prevalence of anemia in people older than 15 years of age is 17.5% with female participants having almost double the prevalence (22.0%) when compared with males (12.2%) (Shisana et al., 2014).

5 Mageu is a traditional fermented drink made from grains. It is also known as amarewu, mageu, mabhundu, and mapotho (See https://www.indemandmarketing.co.za/instant-sorghum-mageu-drink).
